# COVID-19 and non-communicable diseases in complex vulnerable populations: evidence from Jordan

**DOI:** 10.1017/S1463423622000731

**Published:** 2023-01-20

**Authors:** Saverio Bellizzi, Nazeema Muthu, Yousef Khader, Hala Boukerdenna, Dana Darwish, Ala’a Al-Sheikh, Alessio Santoro, Alvaro Alonso-Garbayo

**Affiliations:** 1 Jordan WHO Country Office, Amman, Jordan; 2 Department of Community Medicine, Public Health and Family Medicine, Jordan University of Science and Technology, Irbid, Jordan

**Keywords:** COVID-19, Jordan, migrants, Non-communicable diseases, refugees, vulnerable

## Abstract

More than three in 10 people living in Jordan are immigrants, with the majority being Palestinian and Syrian refugees, who have a very similar non-communicable diseases (NCDs) profile to the hosting Jordanian community. We conducted a rapid review of the literature of studies, reports, and documents on the evidence of the impact of COVID-19 on vulnerable populations in Jordan with regard to NCD during the first year of the pandemic. COVID-19-related mobility constraints and often lack of awareness of NCDs put additional burden on vulnerable populations like refugees and migrants, in particular on non-registered migrants. COVID-19 pandemic and associated mitigation measures led to disruption in routine health services, significantly impacting people living with NCDs. Ensuring to deliver a people-centered and inclusive approach that works well during COVID-19 is of paramount importance toward Universal Health Coverage (all people have access to the health services they need, when and where they need them, without financial hardship).

## Introduction

Since the onset of the COVID-19 pandemic, Jordanian government directed the efforts to manage the impact on the health care system and keep the flow of routine services through supporting primary health care (PHC) and enhancing its capability. At an early stage of the public health emergency, the government prepared protocols regarding mechanisms for dealing, including isolation, with infected people, in addition to establishing fixed COVID-19 testing stations in health centers and hospitals affiliated to the MoH (Al-Tammemi, 2020). Public PHC centers continued to provide the essential services of surveillance, emergency and first aid, mental health, immunization, maternal and child health, which helped many of the community members to receive the care they needed. However, the continuity of quality health services was disrupted: older patients and patients with specific types of diseases and disabilities were particularly affected by the COVID-19 regulations and disruption of health services. For instance, patients with non-communicable diseases (NCDs), such as diabetes or cardiac problems, were impacted by the interruption of their regular visits and the provision of medications, which affected the management of their condition. This was indirectly confirmed by a study, which indicated a 21% increase in standardized mortality in April–December 2020 compared with the same months in the pooled period of 2016–2019, with higher excess deaths occurring among men and in people aged 60 years or older (Khader and Al Nsour, 2021). Besides, Jordan’s NCDs index is already low and delivering medical care for those patients during curfews and lockdowns was critical, because of the serious complications those patients could develop, which results in a higher burden on the health care system on the long-term and lower indices in the Universal Health Coverage (UHC) (WHO, 2021); the latter allows tracing health service coverage for people when and where they need them, without financial hardship, and also, to mention how the significant psychosocial and economic hardship triggered life-threatening acute cardiovascular events among individuals not infected with the virus (Hammoudeh *et al.*, 2021).

Jordan has historically been an important recipient of migrants, which nowadays represent more than one-third of the total population according to the International Organization for Migration. While the United Nations Children’s Fund estimates that the total number of Syrian refugees is around 1.3 million, with most living out of camps, more than 2 million registered Palestine refugees are present (Bellizzi *et al.*, 2021).

In Jordan, United Nations Development Programme (UNDP) reported that 69.3% of the population faced challenges in access to health care during the pandemic and more than 70% referred having difficulties in covering basic needs including food and medicines (UNDP, 2020). Literature has also well documented the synergic role of underlying NCD on COVID-19-related mortality in countries such as in Italy, where COVID-19 deaths in hospitals comprised 68% hypertension and 31% type 2 diabetes (WHO, 2020). Reported data from 21 European countries indicated that only 7.3% of overall COVID-19 deaths were not reported to have co-morbidities (ECDC, 2020). More than 150 million people are living with NCDs in the WHO Eastern Mediterranean Region (EMR); these persons are all at higher risk of developing severe symptoms of COVID-19 due to their preconditions (Slama *et al.*, 2018).

## Jordan NCD context

In line with the global trend, Jordan is increasingly affected by an epidemiological transition toward NCDs which are currently accounting for 78% of all deaths (Jordan Ministry of Health *et al.*, 2019); at the same time, rate of specific risk factors for NCD in Jordan has also drastically increased in 10 years duration (Jordan Ministry of Health, Center for Strategic Studies, USAID, WHO. Jordan National Stepwise (STEPs) for Noncommunicable Diseases Risk Factors, 2019, 2019). The 2019 STEPs survey in Jordan showed that four out of 10 people (41%) among the age group of 18–69 years had three or more NCDs risk factors. The rate of impaired blood glucose or raised blood glucose was estimated at 14% of the overall population, which is comparable to the STEPs results from other neighboring countries like the Occupied Palestinian Territories (OPT) and Lebanon and other countries in the region such as Sudan (Jordan Ministry of Health *et al.*, 2019). Particularly, the findings of the 2019 STEPs alarmingly reported Jordan as one of the highest rates of tobacco smoking in the world that has drastically increased substantially over the past 12 years reaching 66% among males and 17% among females in 2019 (Jordan Ministry of Health *et al.*, 2019). When stratifying by nationality, no significant differences in prevalence of NCDs or of three or more of their risk factors between Jordanians and Syrians were detected (Doocy *et al.*, 2015).

Pre-COVID-19 already high burden of cardiovascular disease and multi-morbidity during/post-COVID-19 inability to access routine care (17% of children under 5 years of age did not receive their basic vaccinations and 23% did not get an access to medical attention for illnesses) may have worsened this already substantial burden.

## Methods

In view of the above, we examined the literature on the evidence of the impact of COVID-19 on vulnerable populations in Jordan, including migrants and refugees, with regard to NCDs. Specifically, we conducted a rapid review of the literature of studies, reports, and documents on the evidence of the impact of the first year of COVID-19 on vulnerable populations in Jordan with regard to NCD; therefore, we did not conduct any statistical analysis. Conversely, we used secondary data and available evidence on the burden of NCDs in Jordan across vulnerable populations and in comparison with other EMR countries.

The literature search was conducted using MEDLINE (including Epub ahead of print, in process and other non-indexed citations), CINAHL, and EMBASE. Additional sources for information included the websites of Global Organizations, National Governments, and National/International Associations. The search terms ‘non-communicable diseases’, ‘chronic illnesses’, ‘migrants’, ‘refugees’, ‘vulnerable populations’, and ‘health system’ were applied and complemented with the terms ‘COVID-19’ and ‘Jordan’.

The authors read all included studies in detail to extract pertinent data for the review using a predesigned extraction sheet. Data extracted included variables, such as names of author(s), organization, sample size, participant selection, and findings presented for three specific domains: access to health care, financial hardship, and awareness.

Data and information generated from reviewing the literature were aggregated, thematically analyzed by taking into consideration Syrian refugees (most of available literature is on this category of refugees), migrants in general, and host population.

## Results

### Access to health care

The current COVID-19 pandemic in Jordan has added stress on the health system that already faced a substantial overload due to mass inflow of refugees in the past years. However, Jordan introduced strong public health measures since the beginning of COVID-19 pandemic to control the pandemic within the constraints imposed by the existing increased demand (Alqutob *et al.*, 2020). Specifically, a national lockdown ordered to ensure complete country isolation through closure of all borders and isolation of administrative governorates from each other. This consequently affected the access to health facilities by some vulnerable populations. Moreover, major tertiary hospitals were exclusively allocated for the treatment of COVID-19 suspending all routine health care which has seriously constrained the overall tertiary care capacity of the health system.

### Financial hardship

Despite the availability of free or subsidized primary health services, the cost of treatment for NCDs was already reported as the primary obstacle to accessing health care by Syrian refugees in Jordan primarily due to the cost of medicines which were out of stock at the point of delivery (Akik *et al.*, 2019). Similarly, over half of Syrian refugees diagnosed with NCDs living outside camps who did not seek care cited provider costs as a barrier (Akik *et al.*, 2019).

Few months into the pandemic, UN agencies and other partners reported a significant impact of COVID-19 on the economic situation of vulnerable populations that led poor accessibility to medications, thereby increasing their risk of health complications when contracting the virus. A rapid assessment of the impact of COVID-19 conducted by the International Labour Organization (ILO) in May 2020 revealed that a third of surveyed Syrians who were in employment before the crisis had lost their jobs permanently, compared to 17% of surveyed Jordanians (ILO & FAFO, 2020). In an UNDP survey, a huge discrepancy was reported between governorates with the pandemic financially impacting urban areas more significantly (UNDP, 2020). In March 2020, the average monthly wages for both surveyed Jordanian and Syrian workers were reduced by more than 40%, and this decline was reported to have been due to reduced working hours as well as the dismissal of some workers from their jobs on a permanent basis (ILO & FAFO, 2020). In terms of health-related vulnerability, one assessment conducted jointly by UNICEF, UNHCR, and WFP revealed that 17% of households had a member with a chronic illness with 30% of them having in addition a disability which increased substantially their vulnerability and constraints to access services (UNICEF *et al.*, 2020).

### Awareness

Despite the fact that various health services are provided to vulnerable population, including those in hard-to-reach areas, by non-governmental organizations and other non-state actors for free, COVID-19-related mobility restrictions put additional burden on vulnerable populations like refugees and migrants, in particular on non-registered migrants. It is also noted that these populations often have less access to health promotion activities which lead to less awareness about how to identify and seek care for NCDs.

COVID-19 pandemic and associated mitigation measures led to disruption in routine health services, significantly impacting people living with NCDs. This disruption is likely to disproportionally burden the most deprived such as migrants and vulnerable host communities. For instance, while more privileged communities relied on teleconsultation and private health care providers, vulnerable communities have much less limited alternatives leading to widening inequality.

## Discussion

Our rapid literature review tried to extrapolate main findings around the burden of NCDs in Jordan during the first year of the COVID-19 pandemic and highlighted the deepened vulnerability of already disadvantaged populations like migrants and refugees.

Such findings are not surprising: according to a modeling exercise published in the 2020 (The Lancet, 2020), two out of 10 people globally are at increased risk of severe COVID-19 mostly due to the coexistence of underlying NCDs) Concurrently, 2020 has witnessed enormous efforts to counteract the coronavirus pandemic, including public health measures, which have led to the disruption of regular health care services in nearly one out of three countries in the world (WHO, 2020). Literature provides strong evidence of the effect of the disruption of NCD services on morbidity due to COVID-19. While in the Netherlands, the number of people newly diagnosed with cancer dropped by 25% as a result of the lockdown, in India 30% fewer acute cardiac emergencies reached health facilities in rural areas in March 2020 compared to the previous year (WHO, 2020). Similarly, cardiac diseases-related Emergency Department (ED) visits dropped by around 35% during the pandemic in the UK. Non-attendance to ED was estimated to cause 84–232 excess cardiac deaths weekly (Katsoulis *et al*., 2021), clearly indicating a collateral damage caused by the COVID-19 pandemic.

While we provide a concise synthesis of the available literature, our report includes various limitations: there may be potential bias in results as household may have provided responses in hopes of securing additional assistance. The likelihood of this bias is potentially high with data collected in a very challenging time with many facing limited access to services, income sources, and livelihoods. Potential Recall Bias is also noted as several questions administered as part of surveys are built upon recall methodologies. This may potentially bias results as respondents may have mis-remembered or mis-represented the actual conditions.

During the recent 67th WHO Regional Committee for EMRO, the WHO EMR NCD Alliance has issued a joint statement that urges Member States to integrate NCD prevention and control into COVID-19 preparedness and response plans (WHO, 2020).

Focusing on general management of NCDs during COVID-19 would be crucial to minimize severe morbidity among refugees and other vulnerable populations during and post-COVID-19 crises. Prioritizing funds on NCD management by international players with an efficient stakeholder engagement strategy is of paramount importance to reach every person suffering from chronic illness. This could therefore enhance the health promotion interventions in the three main settings recommended by WHO: schools, workplaces, and PHC settings.

Ensuring to deliver a people-centered and inclusive approach that works well during COVID-19 is of paramount importance toward UHC. WHO’s guidance on maintaining essential health services during COVID-19 therefore recommends focusing on five approaches to maintain the chronic disease management: (i) increasing awareness on COVID-19 complications among NCD patients, (ii) ensuring accessibility of telehealth or online services, (iii) developing community-based self-management plan, (iv) increasing home supplies of medication, and (v) modifying frequency and means of delivery of routine clinical reviews. To do so, the involvement of civil society and affected communities, including those in humanitarian settings, is key for decision-making and monitoring processes.

On the other hand, potential differences in health between migrants and the host population indicate that lifestyle-related factors play a major role; thus, there is need of population-based approaches to facilitate healthy behavior. In this regard, additional studies on the role of migration and the role of the context would help target more specific prevention strategies.

Finally, we cannot downplay that the ability for Jordan to face such challenges will depend more than ever on the support of the international community, the government, and international institutions supported by the humanitarian sector in the forefront is significant for maintaining stability and ensuring the welfare of the most vulnerable.

## Conclusions

The current COVID-19 pandemic in Jordan has added stress on the health system that already faced a substantial overload due to mass inflow of refugees in the past years. Particularly, the existing NCD burden across vulnerable populations, such as refugees, migrants, and less-advantaged host communities, has been exacerbated due to the compounded effects of limited access to health care, awareness, and the financial barriers created by COVID-19. People-centered and inclusive approach is critical to build back better and ensure appropriate preparedness and readiness of the health system in future public health emergencies.

## References

[ref1] Akik C , Ghattas H , Mesmar S , Rabkin M , El-Sadr WM and Fouad FM (2019) Host country responses to non-communicable diseases amongst Syrian refugees: a review. Conflict and Health 13, 8. doi: 10.1186/s13031-019-0192-2 30949232 PMC6431037

[ref2] Alqutob R , Al Nsour M , Tarawneh MR , Ajlouni A , Khader Y , Aqel I , Kharabsheh S , Obeidat N (2020) COVID-19 Crisis in Jordan: response, scenarios, strategies, and recommendations. JMIR Public Health and Surveillance 6, e19332. doi: 10.2196/19332 32407289 PMC7381018

[ref3] Al-Tammemi AB (2020) The battle against COVID-19 in Jordan: an early overview of the Jordanian experience. Frontiers in Public Health 8. 10.3389/fpubh.2020.00188 PMC7220996 32574291

[ref5] Bellizzi S , Aidyralieva C , Alsawhala L , Al-Shaikh A , Santoro A and Profili MC (2021) Vaccination for SARS-CoV-2 of migrants and refugees, Jordan. Bull World Health Organ 99, 611. doi: 10.2471/BLT.21.285591 34475595 PMC8381092

[ref6] Doocy S , Lyles E , Roberton T , Akhu-Zaheya L , Oweis A and Burnham G (2015) Prevalence and care-seeking for chronic diseases among Syrian refugees in Jordan. BMC Public Health 15, 1097. 26521231 10.1186/s12889-015-2429-3 PMC4628338

[ref7] ECDC (2020) Coronavirus disease 2019 (COVID-19) in the EU/EEA and the UK– ninth update: ECDC. Retrieved from https://www.ecdc.europa.eu/sites/default/files/documents/covid-19-rapid-risk-assessment-coronavirus-disease-2019-ninth-update-23-april-2020.pdf

[ref8] Hammoudeh AJ , Madanat E , Tabbalat R , Ibdah R , Makhamreh H , Fakhri M , Khader Y , Mansour O , Alhaddad IA (2021) Acute cardiovascular events triggered by the COVID-19 pandemic-related stress in non-infected individuals. The Jordan COVID-19 Acute Cardiovascular Events (JoCORE) study. Reviews in Cardiovascular Medicine 22, 1677–1683. doi: 10.31083/j.rcm2204175 34957810

[ref9] ILO & FAFO (2020) Facing Double Crises. Rapid assessment of the impact of COVID-19 on vulnerable workers in Jordan: ILO & FAFO. Retrieved from https://www.ilo.org/beirut/publications/WCMS_743391/lang--en/index.htm

[ref10] Jordan Ministry of Health, Center for Strategic Studies, USAID, WHO. Jordan National Stepwise (STEPs) for Noncommunicable Diseases Risk Factors 2019 (2019) Jordan Ministry of Health. Retrieved from https://www.moh.gov.jo/ebv4.0/root_storage/en/eb_list_page/stepwise_survey_(steps)_2020_technical_report-english.pdf

[ref11] Katsoulis M , Gomes M , Lai AG , Henry A , Denaxas S , Lagiou P , Nafilyan V , Humberstone B , Banerjee A , Hemingway H , Lumbers RT (2021) Estimating the effect of reduced attendance at emergency departments for suspected cardiac conditions on cardiac mortality during the COVID-19 pandemic. Circulation: Cardiovascular Quality and Outcomes 14, e007085. doi: 10.1161/CIRCOUTCOMES.120.007085 33342219 PMC7819531

[ref12] Khader Y and Al Nsour M (2021) Excess mortality during the COVID-19 pandemic in Jordan: secondary data analysis. JMIR Public Health & Surveillance 7, e32559. 34617910 10.2196/32559 PMC8500348

[ref13] Slama S , Lee J , Aragno M , Laroche S and Hogerzeil H (2018) The development of the noncommunicable diseases emergency health kit. Eastern Mediterranean Health Journal 24, 92–8. doi: 10.26719/2018.24.1.92 29658625

[ref14] The Lancet (2020) COVID-19: a new lens for non-communicable diseases. Lancet 396, 649. Erratum in: Lancet. 396:818. Medline:32891195. doi: 10.1016/S0140-6736(20)31856-0 32891195 PMC7470688

[ref15] UNDP (2020) COVID-19 impact on households in Jordan: a rapid assessment: UNDP. Retrieved from https://reliefweb.int/sites/reliefweb.int/files/resources/77030.pdf

[ref16] UNICEF, UNHCR, WFP (2020) Multisectoral rapid needs assessment: COVID-19- Jordan. Retrieved from https://www.unicef.org/jordan/reports/multi-sectoral-rapid-needs-assessment-covid-19-jordan

[ref17] World Health Organization (2020) EMR NCD Alliance has taken part in the 67th WHO Regional Committee for EMRO. NCD Alliance: WHO. Retrieved from https://ncdalliance.org/news-events/news/emr-ncd-alliance-has-taken-part-in-the-67th-who-regional-committee-for-emro

[ref18] World Health Organization (2020) Preliminary results: rapid assessment of service delivery for noncommunicable diseases during the COVID-19 pandemic: WHO. Retrieved from https://www.who.int/publications/m/item/rapid-assessment-of-service-delivery-for-ncds-during-the-covid-19-pandemic

[ref19] World Health Organization (2020) The impact of the COVID-19 pandemic on noncommunicable disease resources and services: results of a rapid assessment: WHO. Retrieved from https://www.who.int/publications/i/item/ncds-covid-rapid-assessment

[ref20] World Health Organization (2021) Tracking universal health coverage: 2021 global monitoring report 2021. Retrieved from https://www.who.int/publications/i/item/9789240040618

